# Integrated Bacterial Community and Differential Metabolites Reveal the Impact of Growth Stage on the Quality of Oat Silage

**DOI:** 10.3390/microorganisms14030516

**Published:** 2026-02-24

**Authors:** Jiahui Ren, Lei Han, Xiaoyun Ma, Xiaoming Liu, Yinglu Hao, Jirui Yuan, Ziyao Ding, Xiaoting Li, Jingyu Wang, Juanjuan Sun

**Affiliations:** 1 Institute of Grassland Research of Chinese Academy of Agricultural Sciences, Hohhot 010010, China; 2 Northern Agriculture and Livestock Husbandry Technology Innovation Center of CAAS, Hohhot 010010, China; 3 National Center of Pratacultural Technology Innovation (Under Preparation), Hohhot 010060, China; 4 Hinggan League Academy of Agricultural and Animal Husbandry Sciences, Ulanhot 137400, China

**Keywords:** oats, fermentation quality, microbial communities, nutrient preservation, metabolic pathway

## Abstract

Growth stage alters substrate availability and moisture in oats, potentially driving microbial succession and metabolite formation during ensiling. Oats were harvested at flowering (FS), milk ripening (MS) and wax ripening (DS) stages and vacuum-bag ensiled for 120 d (*n* = 4 per stage). We measured chemical composition and fermentation products, enumerated key microbes, profiled bacterial communities by 16S rRNA gene amplicon sequencing, and characterised metabolites by untargeted LC–MS. With advancing growth stage, dry matter (DM), neutral detergent fibre (NDF) and acid detergent fibre (ADF) increased, whereas crude protein (CP) decreased. MS silage had the lowest pH (4.63) and the highest CP, whereas FS showed higher lactic acid, but the butyric acid (BA) and NH_3_-N/TN were also significantly higher than those at MS and DS (*p* < 0.001). Community composition shifted from Enterobacter (FS) toward Lactobacillus enrichment (DS), accompanied by stage-specific metabolite signatures (biogenic amines and indoleacetic acid at FS; betaine and purine metabolites at MS; reduced amines and more amino-acid derivatives at DS). Overall, under the conditions of this study, considering fermentation stability, nutrient preservation, microbial diversity, and metabolic pathways, the best balance was achieved during MS.

## 1. Introduction

Oats (*Avena sativa* L.) belong to the genus Avena within the Poaceae family. As a dual-purpose crop for both grain and fodder, they exhibit strong adaptability, including tolerance to cold, drought, poor soils, and saline–alkaline conditions [1]. Oat grains serve as concentrated feed for livestock, while its fresh and dried forage constitute vital roughage sources. Stems and leaves can be processed into hay bales, straw meal, or ensiled. Owing to their tender texture, palatability, and rich nutritional profile, they possess high forage value [2]. As one of the important preservation methods, silage relies on microbial fermentation to rapidly lower the pH of the raw materials. A sufficiently low acidity threshold can prevent the activity of undesirable microorganisms from producing inhibitory, toxic, foul-smelling, or other harmful metabolites, thereby maximising the preservation of the nutritional value of forage and extending its shelf life [3,4]. It can provide stable nutrition to ruminants during periods of fresh forage shortage. However, multiple studies indicate that silage quality is highly sensitive to the growth stage of the raw material [5,6,7]. As growth progresses, dry matter (DM), neutral detergent fibre (NDF), and acid detergent fibre (ADF) typically increase, while crude protein (CP) and water-soluble carbohydrates (WSC) decrease or undergo redistribution. Grain hardening and stem cell wall maturation enhance stem rigidity, reducing feed compactability and impeding rapid establishment of anaerobic conditions within the silo [8]. These changes reshape microbial ecology and metabolic pathways during anaerobic fermentation, thereby influencing key indicators such as lactic acid (LA), butyric acid (BA), and ammonia nitrogen/total nitrogen (NH_3_-N/TN), alongside palatability. In practice, selecting an appropriate harvest stage is a key decision because it simultaneously changes moisture, fermentable carbohydrate availability, and buffering capacity, which together shape fermentation outcomes and nutrient preservation during ensiling.

The quality of silage feed largely depends on the composition and metabolic activity of microbial communities during fermentation [9]. This community is driven not only by substrate supply and moisture content but also influenced by cell wall maturity and osmotic pressure changes associated with growth stages. During early ensiling, epiphytic facultative anaerobes consume residual oxygen and soluble substrates; as pH declines, lactic acid bacteria (LAB) generally become dominant. Lactic acid bacteria (LAB) primarily convert water-soluble carbohydrates into lactic acid, acetic acid, and other products, thereby lowering the pH and inhibiting spoilage microorganisms. When fermentable sugars are insufficient or moisture is excessive, undesirable microorganisms can proliferate, promoting proteolysis and amino acid deamination/decarboxylation, which increases NH_3_-N and may generate biogenic amines and other off-flavour metabolites [10,11]. Methodologically, microbial ecology can be characterised by 16S rRNA amplicon sequencing, and LEfSe (linear discriminant analysis effect size) can identify indicator taxa across multiple groups [12]. Metabolomics bridges ‘microbes–metabolism–phenotype’ by capturing shifts in amino acid, carbohydrate, nucleotide, and vitamin pathways [13,14]. Although several studies have examined maturity effects on oat or small-grain silages [5,6,7,15,16], an integrated multi-omics comparison across multiple oat growth stages within a single experimental framework remains limited. This study addresses that gap by jointly linking substrate changes, bacterial succession, and metabolite signatures to fermentation endpoints.

Therefore, the general objective of this study was to determine how oat growth stage influences silage fermentation quality and nutrient preservation through changes in bacterial community structure and metabolite profiles. Specifically, we compared oats harvested at flowering (FS), milk ripening (MS), and wax ripening (DS) stages by integrating chemical composition, fermentation indices, 16S rRNA gene amplicon sequencing, and untargeted LC–MS metabolomics to (i) characterise stage-dependent physicochemical and microbial differences between fresh forage and silage; (ii) identify key bacterial taxa and metabolic markers associated with fermentation phenotypes; and (iii) evaluate the harvest stage that best balances fermentation stability and nutritional value. We hypothesised that advancing growth stage would alter fermentable substrate availability and moisture, driving shifts in bacterial succession and metabolite formation and thereby producing distinct fermentation outcomes among FS, MS, and DS silages.

## 2. Materials and Methods

### 2.1. Experimental Design

The trial site was located at the Agro-Pastoral Transition Zone Experimental Base of the Grassland Research Institute, Chinese Academy of Agricultural Sciences (Shalqin Town, Tumed Left Banner, Hohhot City, Inner Mongolia; E 111°45′–111°47′, N 40°34′–40°35′, elevation 1044 m). The region exhibits a temperate continental climate with an annual mean temperature of 6.3 °C, a frost-free period of approximately 133 days, and annual precipitation ranging from 300 to 350 mm. The oat variety tested was “Aiwo”, employing a randomised block design. On 24 April 2023, manual furrow sowing was conducted in three plots, each measuring 15 m^2^ (3 m × 5 m) with 12 rows spaced 25 cm apart and 50 cm between plots. The seeding rate was 150 kg·ha^−1^. Prior to sowing, compound fertiliser (total nutrients N-P_2_O_5_-K_2_O > 51%) was applied at a rate of 300 kg·ha^−1^. No additional fertiliser was applied during the growing season, and conventional field management practices were followed after emergence.

### 2.2. Silage Preparation

Fresh forage was harvested at flowering stage (FS), milk ripening stage (MS), and wax ripening stage (DS), promptly transported to the laboratory, and chopped into 2–3 cm segments using a chopper (Xiangdong Machinery Manufacturing Co., Ltd., Loudi, China). Stems, leaves, and panicles were thoroughly mixed before filling polyethylene feed bags, which were vacuum sealed. Each bag contained approximately 300 g, with four independent vacuum-sealed replicates established for each growth stage (*n* = 4). In total, 12 silage bags were prepared (3 stages × 4 replicates); each bag was treated as one biological replicate. After 120 days of storage at room temperature, bags were opened for sampling, and nutritional components and fermentation characteristics were determined.

### 2.3. Analysis of Chemical Composition and Fermentation Characteristics

Raw materials and silage samples were dried to constant weight in a ventilated oven at 65 °C. Dry matter (DM) content was calculated based on fresh weight. After grinding the dry samples, nutritional components were analysed. Crude protein (CP) content was determined using an automatic Kjeldahl apparatus (Model: K1160, Hanon Future Technology Group Co., Ltd., Jinan, China), while neutral detergent fibre (NDF) and acid detergent fibre (ADF) content were measured with an automatic fibre analyser (Model: F2000, Hanon Future Technology Group Co., Ltd., Jinan, China), following standard procedures of Van Soest et al. [17]. Water-soluble carbohydrates (WSC) were determined using the anthrone-sulphuric acid method [18], while crude ash (Ash) content was measured via high-temperature combustion [19].

Ten grams of silage sample were added to 90 mL sterile water and homogenised using a paddle-type sterile homogeniser (Model: JX-05, Shanghai Jingxin Industrial Development Co., Ltd., Shanghai, China). The silage extract was obtained by filtration through four layers of gauze, and its pH was measured using a pH metre (Model: LAQUAtwin-pH-22, Horiba Advanced Techno Co., Ltd., Kyoto, Japan). The extract was filtered through a 0.22 μm membrane for the determination of lactic acid (LA), acetic acid (AA), propionic acid (PA), butyric acid (BA), and ammoniacal nitrogen (NH_3_-N). Organic acid content was determined by high-performance liquid chromatography (K2025HPLC, Shodex Rspark KC-811 column, 8 mm × 300 mm, Diode array detector, Wooking Scientific Instruments Co., Ltd., Shanghai, China), while ammoniacal nitrogen was measured using the phenol-sodium hypochlorite colorimetric method [20].

The microbial count in the silage extract was determined by the plate count method after appropriate dilution. Lactic acid bacteria (LAB) were cultured anaerobically at 37 °C for 72 h on MRS medium [21] (Guangdong Huankai Microbial Technology Co., Ltd., Guangzhou, China). Yeast and moulds were cultured at 30 °C for 72 h on malt extract agar medium (Guangdong Huankai Microbial Technology Co., Ltd., Guangzhou, China). Escherichia coli was cultured aerobically at 37 °C for 24 h on crystal violet-carmine bile salt glucose agar [22] (Guangdong Huankai Microbial Technology Co., Ltd., Guangzhou, China). Following incubation, all microbial species underwent plate counting, with microbial quantities calculated via dilution factors.

### 2.4. DNA Extraction, PCR Amplification and Sequence Processing

Total DNA was extracted using the HiPure Soil DNA Kit (Magen, Guangzhou, China). Amplification of the 16S rRNA V3–V4 region (primers 341F: CCTACGGGNGGCWGCAG; 806R: GGACTACHVGGGTATCTAAT): 95 °C for 5 min; followed by 30 cycles (95 °C for 1 min, 60 °C for 1 min, 72 °C for 1 min); final extension at 72 °C for 7 min. The 50 μL reaction mixture included 10 μL 5 × Q5 Reaction Buffer, 10 μL 5 × Q5 High-GC Enhancer, 1.5 μL 2.5 mM dNTPs, 1.5 μL each of forward/reverse primers (10 μM), Q5 High-Fidelity DNA Polymerase 0.2 μL, template DNA approximately 50 ng (New England Biolabs, South San Francisco, CA, USA). Amplification quality was assessed via 2% agarose gel electrophoresis, purified using AMPure XP Magnetic Beads (Beckman Coulter, Brea, CA, USA), and quantified by Qubit 3.0. Library preparation was conducted using the Illumina DNA Prep Kit (Illumina, San Diego, CA, USA), with quality control performed on the ABI StepOnePlus. Qualified libraries were sequenced on the Illumina NovaSeq 6000 (PE250) platform by Gene Denovo Biotechnology Co., Ltd. (Guangzhou, China).

Raw paired-end sequencing reads were processed using the standard amplicon workflow, which includes quality filtering, paired-end merging, and removal of chimeric sequences. Taxonomic assignment was performed against 16S rRNA reference database. Alpha diversity indices and beta diversity distances were calculated based on the processed feature table. Differential taxa were identified using LEfSe as described in Section 2.6.

### 2.5. Metabolomics Analysis

Approximately 100 mg of liquid nitrogen–ground silage sample was transferred to a 1.5 mL EP tube. An 80% methanol–water solution (500 μL) was added; the mixture was vortexed and then kept on ice for 5 min. Samples were centrifuged at 15,000× *g* for 20 min at 4 °C, and the supernatant was collected and diluted with mass spectrometry–grade water to a final methanol fraction of 53%. After a second centrifugation (15,000× *g*, 20 min, 4 °C), the supernatant was used for LC–MS injection. For liquid samples, 100 μL aliquots were mixed with 400 μL of 80% methanol–water and processed as described above. Untargeted metabolomics was performed by Gene Denovo Biotechnology Co., Ltd. (Guangzhou, China) using a Vanquish UHPLC system coupled to a Q Exactive HF-X mass spectrometer (Thermo Fisher Scientific, Waltham, MA, USA). Metabolites were separated on a Hypersil GOLD column (100 mm × 2.1 mm, 1.9 μm) at 40 °C with a flow rate of 0.2 mL·min^−1^. In positive ion mode, mobile phase A was 0.1% formic acid in water and mobile phase B was methanol; in negative ion mode, mobile phase A was 5 mM ammonium acetate (pH 9.0) and mobile phase B was methanol. Raw data (.raw) were converted to mzXML using ProteoWizard (Palo Alto, CA, USA) and processed in XCMS for peak extraction, alignment, and quantification. Metabolite annotation was performed by matching MS/MS spectra to an in-house library with a mass tolerance of 10 ppm.

### 2.6. Statistical Analysis

Data were organised using Excel and one-way analysis of variance (ANOVA) was performed with SPSS (IBM SPSS Statistics 27.0.1), presented as mean ± standard error of the mean (SEM). Normality (Shapiro–Wilk) and homogeneity of variance (Levene) were tested when necessary, and post hoc comparisons were done using Tukey HSD; significance threshold was set at *p* < 0.05. Microbial community alpha diversity was plotted using GraphPad Prism 10.5.0 software. Relative abundance of community structure, Adonis, Linear Discriminant Analysis Effect Size (LEfSe), Principal Component Analysis (PCA), Principal Co-ordinates Analysis (PCoA) and differential KEGG enrichment were drawn using the Omicsmart system of Gene Denovo Corporation (https://www.omicsmart.com, accessed on 5 December 2025), and Pearson correlation plots were created using Chiplot (https://www.chiplot.online, accessed on 10 December 2025).

## 3. Results

### 3.1. Nutritional Quality of Fresh Oat Raw Material and Silage

The nutritional quality of fresh oat raw material and silage at different growth stages is presented in Table 1. Significant differences were observed in nutritional quality between flowering, milk ripening, and wax ripening stages (*p* < 0.05). As the growth stage progressed, the DM, WSC, NDF, and ADF of the oat raw materials showed a gradual increasing trend with significant differences, with the wax ripening stage being significantly higher than the milk ripening stage and flowering stage (*p* < 0.05); CP showed a gradual decreasing trend, with the flowering stage having the highest CP content. Following fermentation, CP, WSC, NDF, and ADF content in silage from flowering, milk, and wax ripeness stages all exhibited a decreasing trend. WSC decreased markedly, indicating its thorough utilisation during fermentation. Among the silage, milk ripening-stage oats contained the highest CP, while flowering stage NDF and ADF were significantly lower than those in milk and wax ripening stages (*p* < 0.05).

### 3.2. Fermentation Quality and Microbial Counts of Oat Silage

Fermentation quality and microbial counts of oat silage at different growth stages are presented in Table 2. LA content in flowering stage was significantly higher than in both milk ripening and wax ripening stages (*p* < 0.05), while PA in milk ripening stage was significantly lower than in flowering and wax ripening stages (*p* < 0.001). BA and NH_3_-N/TN in flowering stage were significantly higher than in milk and wax ripening stages (*p* < 0.001). Microbial plate count results indicated that LAB, Yeast and Moulds counts in flowering stage were significantly higher than in the other two stages (*p* < 0.05).

### 3.3. Diversity of Microbial Communities in Oat Silage

**Figure 1:**
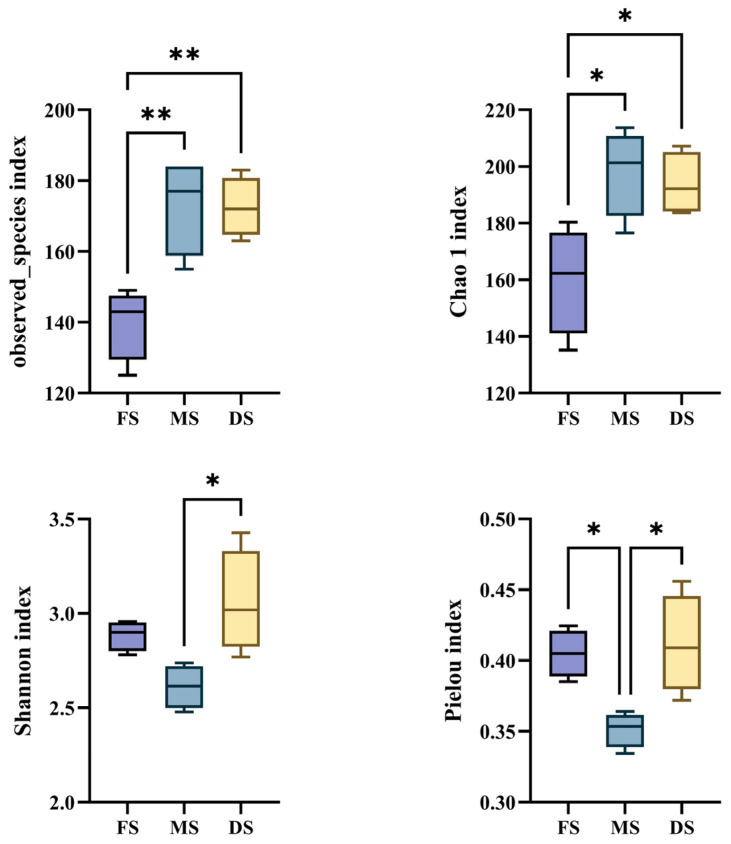
Alpha diversity of microbial community structure. Note: observed_species index: indicates the actual number of OTUs observed; Chao1 index: used to measure the species richness of the sample; Shannon index: reflects both species richness and evenness; Pielou index: measures the evenness of relative species abundance; FS: Flowering stage, MS: Milk ripening stage, DS: Wax ripening stage. Each treatment is repeated 4 times (*n* = 4). Boxes of different colors represent treatments at different growth stages: purple for flowering stage, blue for milk ripening stage, and yellow for wax ripening stage. * *p* < 0.05; ** *p* < 0.01.

Analysis of alpha diversity in microbial community structure (Figure 1) revealed that the observed_species index showed significantly fewer OUTs detected during the flowering stage compared to the milk and wax ripening stages (*p* < 0.01). The Chao1 index indicated significantly fewer species detected during flowering than during milk and wax ripening stages (*p* < 0.05), with the milk ripening stage exhibiting the highest species richness and diversity. The Shannon index revealed that species richness and evenness during the milk ripening stage were significantly lower than during the wax ripening stage (*p* < 0.05). The Peilou index was significantly lower during the milk ripening stage compared to both the flowering stage and the wax ripening stage (*p* < 0.05), indicating that the microbial community during the milk ripening stage was dominated by a small number of species.

**Figure 2:**
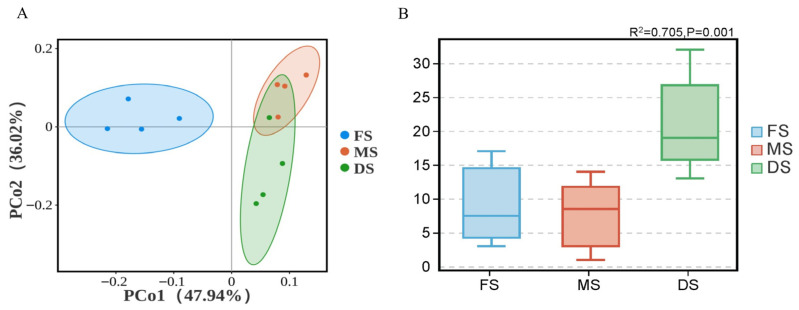
Beta diversity of microbial community structure. Note: (**A**): Principal Co-ordinates Analysis (PCoA) at the OTU level based on Bray–Curtis distance; (**B**): Adonis statistical test at the OTU level based on Bray–Curtis distance; FS: Flowering stage, MS: Milk ripening stage, DS: Wax ripening stage. Each treatment is repeated 4 times (*n* = 4).

Based on Bray–Curtis distance, Principal Co-ordinates Analysis (PCoA) of silage from oats at different growth stages revealed distinct separation trends in microbial community structure between flowering, milk ripening, and wax ripening stages. PCo1 and PCo2 explained 47.94% and 36.02% of the total variance, respectively (Figure 2A). The Adonis statistical test further confirmed that the growth stage factor explained 70.50% of the variation in microbial community structure (R^2^ = 0.705), with significant differences between groups (*p* = 0.001) (Figure 2B). This result indicates that growth stage is a key factor driving the succession of microbial community structure in oat silage.

### 3.4. Microbial Community Composition of Oat Silage

**Figure 3:**
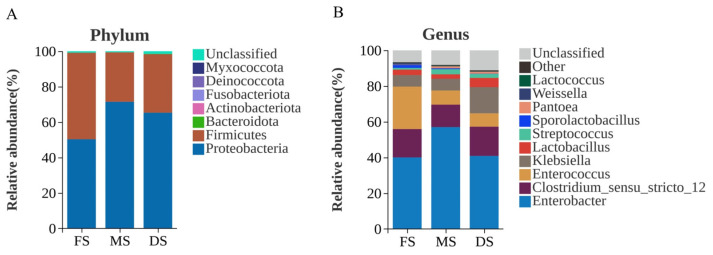
Relative abundance of microbial community structure. Note: (**A**): Relative abundance at the bacterial phylum level, (**B**): Relative abundance at the bacterial genus level; FS: Flowering stage, MS: Milk ripening stage, DS: Wax ripening stage. Each treatment is repeated 4 times (*n* = 4).

As shown in Figure 3, significant differences exist in bacterial community structure among oat silage samples harvested at different growth stages. At the phylum level (Figure 3A), the dominant phyla for flowering, milk ripening and wax ripening stage oat silage were *Proteobacteria* and *Firmicutes*. During the flowering stage, their relative abundances were 50.31% and 48.82%, respectively; during the milk ripening stage, they were 71.44% and 27.89%, respectively; and during the wax ripening stage, they were 65.29% and 33.03%, respectively. As the growth stage progressed, the relative abundance of *Proteobacteria* gradually increased, reaching a maximum value (71.44%) during the milk ripening stage, while the relative abundance of *Firmicutes* gradually decreased, reaching its minimum (27.89%) during the milk ripening stage. At the genus level (Figure 3B), the dominant genera during the flowering stage were *Enterobacter* and *Enterococcus*, with relative abundances of 40.01% and 23.79%, respectively. As the growth stage progressed, *Enterobacter*’s relative abundance increased to 57.01% by the milk ripening stage, while *Enterococcus* decreased to 7.90%. *Enterobacter* remained the dominant genus during the wax ripening stage, while *Klebsiella* and Unclassified maintained relatively high abundances. *Clostridium_sensu_stricto_12* exhibited consistently high relative abundances across all three stages (15.88%, 12.57%, and 16.38%), with the lowest relative abundance observed during the milk ripening stage.

### 3.5. Indicator Species in Oat Silage

**Figure 4:**
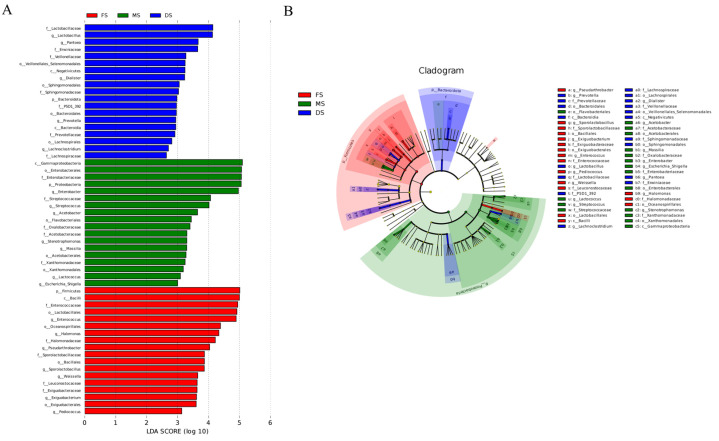
LEfSe analysis of microbial communities based on genus level. Note: (**A**): LDA distribution histogram (*p*-value < 0.05, LDA score ≥ 2), (**B**): Species evolutionary branch diagram, where the radiating circles from inside to outside represent taxonomic levels from phylum to genus; yellow nodes indicate non-significant species, non-yellow nodes indicate significant microbes; FS: Flowering stage, MS: Milk ripening stage, DS: Wax ripening stage. Each treatment is repeated 4 times (*n* = 4).

To identify key microbial markers of oat silage at different growth stages, the bacterial communities in the silage were analyzed using LEfSe (Linear Discriminant Analysis Effect Size) with an LDA score ≥ 2, as shown in Figure 4. Within the bacterial communities, 55 microbial species were identified as significantly different indicator species across the three growth stages. During the flowering stage, *Enterococcus*, *Halomonas*, *Pseudarthrobacter*, *Sporolactobacillus*, *Weissella*, *Exiguobacterium*, and *Pediococcus* were characteristic microorganisms. At the milk ripening stage, *Enterobacter*, *Streptococcus*, *Acetobacter*, *Stenotrophomonas*, *Massilia*, *Lactococcus*, and *Escherichia-Shigella* are identified as primary marker species. By the wax ripening stage, *Lactobacillus* became the most significantly enriched genus, alongside increased abundance of *Pantoea*, *Dialister*, *Prevotella*, and *Lachnoclostridium*.

### 3.6. Correlation Between Bacterial Communities (Top 20 Genera) and Nutritional and Fermentation Characteristics of Oat Silage

**Figure 5:**
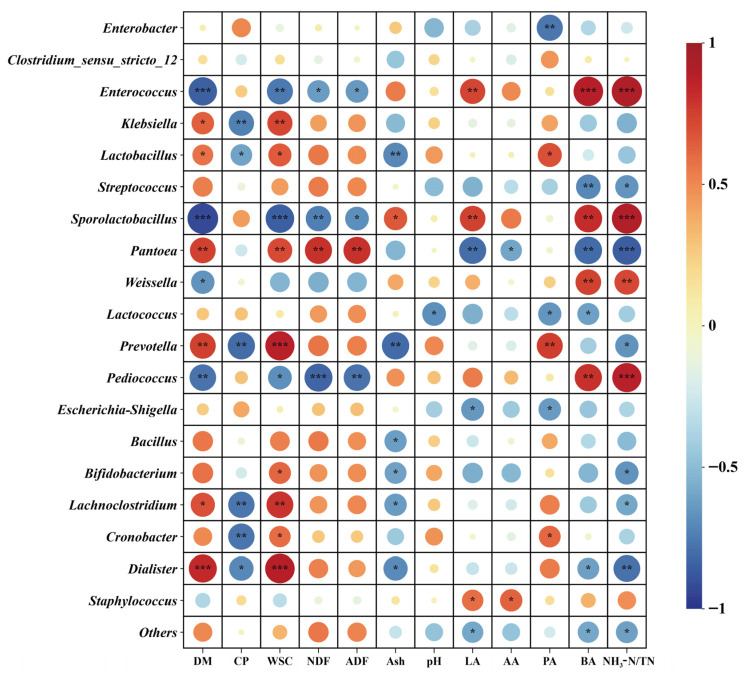
Pearson correlation analysis between bacterial communities (top 20 genera) and oat silage nutrition and fermentation characteristics. Note: R values are represented by different colours in the chart, with red indicating a positive correlation (0 < R < 1) and blue indicating a negative correlation (−1 < R < 0); *p*-values are indicated as follows: * *p* < 0.05, ** *p* < 0.01, *** *p* < 0.001. DM: Dry matter, CP: Crude protein, WSC: Water-soluble carbohydrates, NDF: Neutral detergent fibre, ADF: Acid detergent fibre, LA: Lactic acid, AA: Acetic acid, PA: Propionic acid, BA: Butyric acid, NH_3_-N/TN: Ammoniacal Nitrogen/Total Nitrogen.

Pearson correlation analysis was conducted between the top 20 bacterial genera in oat silage samples and silage nutritional and fermentation physicochemical parameters (DM, CP, WSC, NDF, ADF, Ash, pH, LA, AA, PA, BA, NH_3_-N/TN) (Figure 5). Results indicated strong correlations between microbial abundance and DM, CP, WSC, LA, BA, and NH_3_-N/TN. *Enterococcus* and *Sporolactobacillus* exhibited extremely significant negative correlations with DM (*p* < 0.001), while *Dialister* showed extremely significant positive correlations with DM (*p* < 0.001). *Klebsiella*, *Prevotella*, *Lachnoclostridium*, and *Cronobacter* exhibited significant negative correlations with CP (*p* < 0.01). *Lactobacillus* showed a significant positive correlation with WSC (*p* < 0.05), while *Lactococcus* exhibited significant negative correlations with pH and BA (*p* < 0.05). When these two bacterial groups were dominant, acidification accelerated and butyrate fermentation was inhibited. *Enterococcus* and *Sporolactobacillus* showed significant positive correlations with LA (*p* < 0.01). *Streptococcus* and *Pantoea* exhibited significant negative correlations with BA (*p* < 0.01). *Streptococcus*, *Prevotella*, *Bifidobacterium*, and *Lachnoclostridium* demonstrated significant negative correlations with NH_3_-N/TN (*p* < 0.05). *Lactococcus* exhibited a significant negative correlation with both pH and BA (*p* < 0.05). *Enterococcus* showed a highly significant positive correlation with both BA and NH_3_-N/TN (*p* < 0.001), indicating its activity is associated with enhanced protein ammonification and butyrate fermentation.

### 3.7. Metabolomics Principal Component Analysis

**Figure 6:**
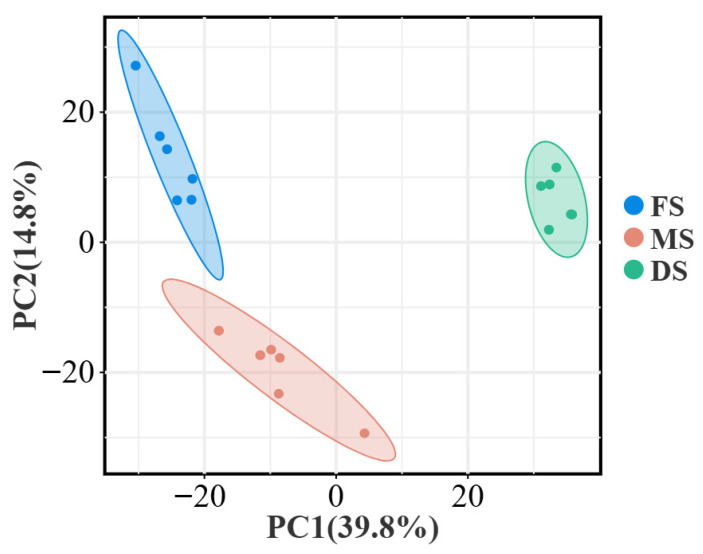
Metabolomic principal component analysis; Note: FS: Flowering stage, MS: Milk ripening stage, DS: Wax ripening stage. Each treatment is repeated 4 times (*n* = 4).

As shown in Figure 6, principal component analysis based on metabolite expression data indicates a clear separation trend among metabolites at the flowering, milk ripening, and wax ripening stages, with good reproducibility within each group. Principal component one (PC1) accounted for 39.8% of the variance in the raw dataset, whilst principal component two (PC2) explained 14.8%. Significant differences were observed across growth stages, indicating that growth stage constitutes a key determinant of metabolic variation in oat silage.

### 3.8. Identification and Analysis of Differential Metabolites

**Figure 7:**
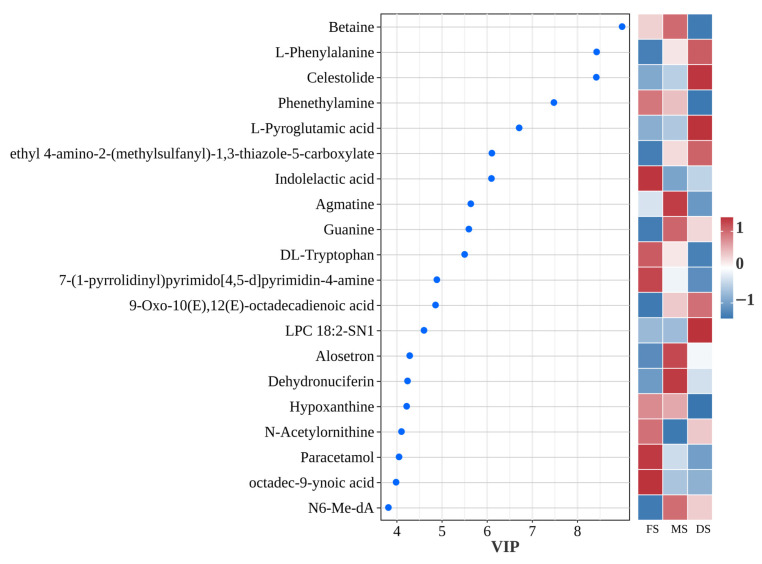
Differential metabolites Variable Importance in Projection (VIP) statistics.The specific numerical statistics can be seen in Table S1. Note: Gradient colors indicate the expression levels of significantly different metabolites at various growth stages. Red represents upregulated metabolite expression, with deeper red indicating higher metabolite abundance; blue represents downregulated metabolite expression, with deeper blue indicating lower metabolite abundance. FS: Flowering stage, MS: Milk ripening stage, DS: Wax ripening stage. Each treatment is repeated 4 times (*n* = 4).

The OPLS-DA-based Variable Importance in Projection (VIP) plot elucidates the significance of the top 20 differential metabolites across three growth stages and their contribution to sample differentiation (Figure 7). From the perspective of metabolic type and direction, the discriminative signals across the three growth stages exhibit a phased pattern transitioning from heterocyclic organic compounds to amino acids and their derivatives. During the flowering stage, metabolites such as indoleacetic acid, paracetamol, and octadec-9-enoic acid exhibited significantly elevated expression levels, while phenethylamine showed markedly increased expression. This indicates that the oat silage was undergoing intense protein degradation and microbial decarboxylation at this stage. At the milk ripening stage, the silage exhibits distinct transitional characteristics such as enhanced lipid metabolism, active osmoprotectant betaine, and significant guanine upregulation, indicating nucleotide turnover and degradation. By the wax ripening stage, biogenic amine accumulation (e.g., phenethylamine and agmatine) diminishes with markedly reduced expression, signalling decreased protein degradation. Amino acids and their derivatives, including L-Phenylalanine, L-Pyroglutamic acid, and N-Acetylornithine, were markedly upregulated, while DL-Tryptophan, a precursor of Indoleacetic acid, was significantly downregulated. This signifies the efficient channelling of tryptophan towards the homolactic fermentation pathway.

### 3.9. Metabolic Pathway Analysis

**Figure 8:**
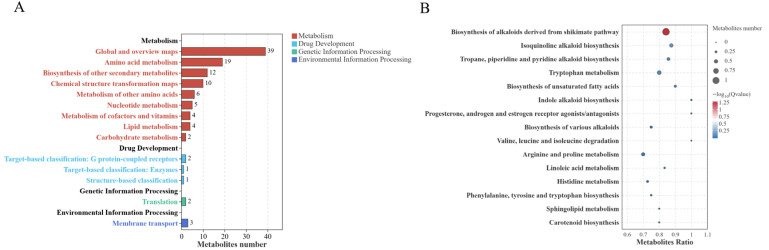
KEGG enrichment analysis of differential metabolites. The quantity statistics can be seen in Tables S2 and S3. Note: (**A**): Statistical chart of the number of differential metabolites; black text represents KEGG-A level classification information, and coloured text represents KEGG-B level classification information. (**B**): Bubble chart of differential KEGG enrichment significance; the size of the bubbles indicates the number of differential metabolites enriched in the pathway, and the colour of the bubbles indicates the enrichment significance in the pathway. Each treatment is repeated 4 times (*n* = 4).

KEGG enrichment analysis based on differential metabolites between flowering, milk ripening and wax ripening stages revealed that enrichment pathways were predominantly dominated by the broad category of Metabolism (Figure 8A). Specifically, the ‘Global and overview maps’ category contained the highest number of metabolites (*n* = 39), followed by ‘Amino acid metabolism’ (*n* = 19), ‘Biosynthesis of other secondary metabolites’ (*n* = 12), and ‘Chemical structure transformation maps’ (*n* = 10). Additionally, enrichment was observed in ‘Metabolism of other amino acids’ (*n* = 6), ‘Nucleotide metabolism’ (*n* = 5), ‘Metabolism of cofactors and vitamins’ (*n* = 4), ‘Lipid metabolism’ (*n* = 4), and ‘Carbohydrate metabolism’ (*n* = 2). Among non-metabolic categories, only minor enrichments were observed, including Drug Development-related entries: Target-based classification: G protein-coupled receptors (*n* = 2), Target-based classification: Enzymes (*n* = 1), Structure-based classification (*n* = 1); Translation within Genetic Information Processing (*n* = 2); and Membrane transport within Environmental Information Processing (*n* = 3). Overall, differences across the three gestational periods primarily manifested as extensive restructuring of metabolic networks, with relatively minor alterations in genetic information processing and membrane transport.

KEGG enrichment analysis revealed alkaloid and aromatic amino acid-related pathways as the most prominent among the top 15 enriched pathways (Figure 8B). Among these, ‘Biosynthesis of alkaloids derived from shikimate pathway’ exhibited the highest enrichment intensity and number of metabolites, while ‘Isoquinoline alkaloid biosynthesis’ and ‘Tropane, piperidine and pyridine alkaloid biosynthesis’ were also significantly enriched. Pathways associated with aromatic amino acid metabolism, such as ‘Tryptophan metabolism’ and ‘Phenylalanine, tyrosine and tryptophan biosynthesis’, also ranked prominently. Regarding lipid metabolism, pathways including ‘Biosynthesis of unsaturated fatty acids’, ‘Linoleic acid metabolism’, and ‘Sphingolipid metabolism’ were enriched, suggesting that the alternation of generations is accompanied by a remodelling of membrane lipids and fatty acid composition. Pathways linked to nitrogen metabolism, such as ‘Arginine and proline metabolism’, and those associated with branched-chain amino acids, including ‘Valine, leucine and isoleucine degradation’, were also selected. Colour-related and antioxidant pathways, notably ‘Carotenoid biosynthesis’, were similarly affected. Most pathways exhibited Metabolites Ratios close to 0.8–1.0, with multi-corrected Q-values reaching statistical significance, collectively pointing to metabolic reorganisation centred on amino acid, alkaloid, and lipid metabolism.

In summary, metabolic differences in oats from flowering through the milk ripening stage to the wax ripening stage primarily concentrate in basal metabolism (particularly amino acid metabolism) and secondary metabolism (multiple alkaloids and carotenoids), accompanied by coordinated regulation of lipid metabolism and membrane transport.

### 3.10. Correlation Between Microorganisms and Differentially Abundant Metabolites

**Figure 9:**
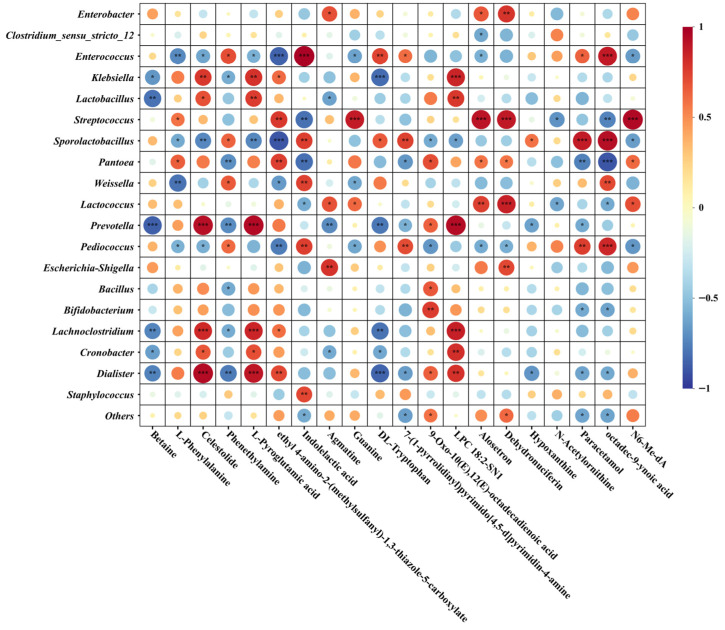
Pearson correlation between microorganisms and differential metabolites (top 20 VIP values). Note: R values are represented by different colours in the chart, with red indicating positive correlation (0 < R < 1) and blue indicating negative correlation (−1 < R < 0); *p*-values are indicated in the chart as follows: * for *p* < 0.05, ** for *p* < 0.01, *** for *p* < 0.001.

As depicted in Figure 9, a significant correlation exists between bacterial communities and differentially abundant metabolites. Fermentation-dominant bacteria represented by *Lactobacillus*, *Streptococcus*, *Sporolactobacillus*, and *Lactococcus* exhibited overall positive correlations with most differentially abundant metabolites. Notably, *Streptococcus* demonstrated a highly significant positive correlation (*p* < 0.001) with certain metabolites. Conversely, *Enterococcus* and *Pediococcus* exhibited negative correlations with most metabolites. *Enterobacter*, *Clostridium sensu stricto 12*, *Escherichia-Shigella*, *Bacillus*, and *Bifidobacterium* demonstrated more differentiated association patterns with metabolites, showing selective correlations with different compounds. Purine/pyrimidine degradation products (Hypoxanthine) exhibited negative correlations with most bacteria. Furthermore, membrane lipid-related molecules (LPC 18:2-SN1) showed positive correlations with numerous bacteria including *Klebsiella* and *Prevotella*, suggesting potential remodelling of lipid/membrane components during fermentation. Betaine, phenethylamine, 7-(1-pyrrolidinyl)pyrimido[4,5 d]pyrimidin-4-amine, paracetamol, and octadec-9-ynoic acid showed negative correlations with most bacteria, including *Klebsiella* and *Lactobacillus*.

## 4. Discussion

### 4.1. Effects of Growth Stage on Nutritional Composition and Silage Fermentation Characteristics of Oat Forage

This study found that as the growth stage of oats progressed, DM, NDF, and ADF increased significantly, whereas CP decreased markedly (Table 1). This pattern aligns closely with the maturation process typical of grass crops [5,7]. Plant maturation involves substantial deposition of structural carbohydrates (e.g., cellulose, hemicellulose, and lignin) within cell walls, leading to elevated NDF and ADF levels. Concurrently, protein within nutritive organs undergoes redistribution or dilution due to reproductive growth prioritisation [23,24]. Notably, WSC content increases with advancing growth stage, peaking at the DS, primarily attributable to starch accumulation in stems and grains [25]. However, following silage fermentation, WSC content decreases substantially across all growth stages, indicating its consumption by microorganisms as a substrate during fermentation—a critical prerequisite for successful fermentation [26].

Silage fermentation quality directly reflects the efficiency of the fermentation process. In this study, FS silage produced the highest concentration of LA yet simultaneously exhibited significantly higher BA and NH_3_-N/TN levels compared to MS and DS (Table 2). This seemingly contradictory outcome reveals the complexity of flowering stage silage fermentation. A higher LA indicates active lactic acid fermentation, which may be related to a higher initial microbial count in the raw materials during the flowering stage [27]. However, high BA and NH_3_-N/TN typically signify undesirable fermentation, indicating BA fermentation and severe protein degradation, respectively [28]. We hypothesise that the lower dry matter content (232.62 g/kg FM) of flowering stage material may have led to increased silage effluent, creating a moist environment conducive to the growth of undesirable microorganisms such as *Clostridia* [11]. Despite high LAB counts, the coexistence of competing microorganisms may have partially converted LA to BA and exacerbated protein deamination. Notably, FS silage showed a higher pH than MS despite higher organic acids because pH reflects the balance between acid dissociation in the aqueous phase and buffering capacity. The higher NH_3_-N/TN in FS indicates greater accumulation of ammonia and amines, which increase buffering capacity and can partially neutralise acids. In addition, BA is a weaker acid than lactic acid, and a greater proportion of BA may contribute less to pH decline than an equivalent amount of lactic acid.

In contrast, MS silage exhibited the optimal balance in fermentation quality. Although its LA content is lower than that of FS silage, it remains at a favourable level, while BA and NH_3_-N/TN are significantly reduced, indicating well-preserved protein and effective inhibition of BA fermentation. This is primarily attributable to the moderate DM content (297.94 g/kg FM) and ample WSC substrate in MS raw material, creating ideal conditions for homolactic fermentation. Silage from the wax ripening stage exhibited the highest DM content. While this effectively suppressed clostridial activity, it may have constrained the compaction density of the silage material, impeding the rapid establishment of an anaerobic environment. Consequently, LA yield was relatively low, and a higher proportion of PA was observed. PA accumulation may be associated with the activity of certain specific microorganisms under conditions of higher DM and residual oxygen, or with heterofermentative lactic acid fermentation pathways [29].

### 4.2. Succession of Microbial Community Structure and Function Driven by Growth Stage

Microbial succession during ensiling is not only a determinant of fermentation outcomes but also a response to raw material properties and the initial epiphytic microbial community, both of which vary with growth stage. 16S rRNA sequencing analysis clearly demonstrates that growth stage is the key driver shaping the microbial community structure of oat silage. PCoA and Adonis analyses revealed significant separation among microbial communities across the three growth stages, primarily attributable to the distinct physicochemical properties of raw materials at different growth stages providing microorganisms with markedly different ecological niches. This substrate-driven community assembly mechanism has been widely demonstrated in silage ecosystems [30,31]. Accordingly, the impact of the growth stage on silage quality is mainly through its upstream effects on the raw materials and the initial attached microorganisms, which in turn determine acidification, nitrogen transformation, and the metabolite profile.

At the phylum level, Proteobacteria and Firmicutes dominated throughout the ensiling process (Figure 3A), consistent with most studies on ensiled forages [32]. As the growth stage progressed, the relative abundance of Proteobacteria increased, while that of Firmicutes reached its lowest point during the MS. This shift may correlate with changes in raw material WSC and DM content. Firmicutes typically exhibit greater competitiveness in WSC-rich environments, whereas certain Proteobacteria demonstrate enhanced adaptability to drier or more complex substrates. Succession becomes more discernible at the genus level (Figure 3B and Figure 4). The FS was dominated by Enterococcus and Enterobacter genera. Enterococcus is generally recognised as a pioneer group in early silage fermentation, capable of rapidly initiating acidification, though its fermentation patterns are diverse and sometimes associated with elevated ammonium nitrogen levels. In this study, Enterococcus exhibited extremely significant positive correlations with BA and NH_3_-N/TN (Figure 5), strongly suggesting its potential involvement in undesirable fermentation processes during the FS silage, rather than purely homolactic fermentation. The microbial community underwent a significant transformation during the MS, with a substantial increase in the relative abundance of Enterobacter. Belonging to the Enterobacteriaceae family, Enterobacter is typically regarded as a ‘conditionally pathogenic bacterium’ or ‘spoilage bacterium’ in silage. Possessing deaminase activity, it may lead to protein loss and ammonium nitrogen accumulation [33]. Nevertheless, in the silage at the MS studied here, despite high Enterobacter abundance, the NH_3_-N/TN ratio remained low. This apparent contradiction may be explained by two mechanisms: firstly, the rapidly declining pH during MS silage fermentation may have inhibited the deaminase activity of most Enterobacter species; secondly, functional redundancy or mutual exclusion within the community may have allowed other microbial activities to compensate for or suppress these detrimental effects. Moreover, the highest Chao1 index was observed during the MS (Figure 1), indicating this period represents a transitional phase of diverse microbial coexistence. Against the backdrop of rapidly established acidification, high diversity does not necessarily lead to poor fermentation; it may instead signify enhanced stability arising from functional redundancy and mutual inhibition. while the rapid pH decline inhibited Enterobacteriaceae deaminase activity, thereby resolving the apparent contradiction of ‘elevated Enterobacter levels but low NH_3_-N/TN ratios during the MS’ [34]. The Shannon and Peilou indices revealed significantly lower species richness and evenness in the milk ripening stage compared to the DS (*p* < 0.05), indicating that the MS microbiome was dominated by a few key species, promoting the dominance of preferred fermentative communities [35].

By the DS, the microbial community exhibited significant changes beneficial to silage quality. LEfSe analysis unequivocally identified Lactobacillus as a key biomarker for the DS (Figure 4). Lactobacillus is recognised as highly efficient homofermentative lactic acid bacteria, capable of producing substantial lactic acid, rapidly lowering pH, and suppressing harmful microorganisms [36]. Their enrichment correlates closely with the higher WSC content in DS raw material, providing ample substrate for their growth. Correlation analysis further demonstrated a significant positive association between Lactobacillus and WSC (Figure 5). Although the final LA concentration in DS silage was not the highest—potentially due to other microbial groups dominating early fermentation or physical constraints (high DM content) limiting its absolute dominance—the rise in Lactobacillus undoubtedly played a crucial role in suppressing BA production and stabilising the silage environment during the later fermentation phase.

### 4.3. Reconstruction of Silage Fermentation Pathways from a Metabolomics Perspective

Non-targeted metabolomics offers a novel perspective for understanding the profound impact of growth stages on silage fermentation. Principal Component Analysis revealed distinct separation of metabolite profiles across the three growth stages (Figure 6), indicating a fundamental restructuring of the metabolic state throughout the fermentation system. The dynamic changes in the top 20 volatile metabolites (VIPs) reveal a distinct metabolic pathway conversion pattern (Figure 7). In silage from the FS, metabolites such as indoleacetic acid and phenethylamine were significantly upregulated. Phenethylamine, a biogenic amine, typically accumulates through microbial decarboxylation of phenylalanine (e.g., by certain enterococci or enterobacteria), serving as an indicator of intense protein degradation [37]. This correlates with the elevated NH_3_-N/TN ratio in FS silage, confirming vigorous proteolysis and amino acid conversion during FS. The MS exhibited distinct metabolic transition characteristics. On one hand, active betaine metabolism suggested microbial adaptation to osmotic pressure shifts [38]; on the other, guanine upregulation reflected nucleotide turnover and degradation, potentially resulting from rapid microbial cell turnover or programmed plant cell death [39]. The DS metabolic profile indicated a trend towards more efficient and orderly fermentation. Accumulation of phenethylamine and agmatine diminishes, while amino acids and their derivatives—including L-phenylalanine, L-pyroglutamic acid, and N-acetylornithine—are significantly upregulated. This shift from biogenic amines to free amino acids indicates a change in the pattern of protein degradation, moving from vigorous deamination/decarboxylation towards milder proteolysis. More importantly, the tryptophan precursor DL-Tryptophan was downregulated during the DS, while its downstream product Indoleacetic acid was highly expressed during FS. This suggests that tryptophan may be more efficiently directed towards alternative metabolic pathways during the DS, such as those associated with Lactobacillus-dominated homolactic fermentation networks.

KEGG pathway enrichment analysis further corroborated these inferences (Figure 8). Pathways associated with amino acid metabolism, such as ‘Tryptophan metabolism’, ‘Arginine and proline metabolism’, and ‘Valine, leucine and isoleucine degradation’, were significantly enriched. Moreover, the highly enriched alkaloid synthesis pathways (e.g., ‘Biosynthesis of alkaloids derived from shikimate pathway’) were particularly noteworthy. Many of these alkaloids are amino acid derivatives; their accumulation not only reflects microbial modification of plant secondary metabolites but may also influence the palatability and bioactivity of silage [40]. The enrichment of lipid metabolism pathways (e.g., ‘Biosynthesis of unsaturated fatty acids’) suggests that the alternation of vegetative phases is accompanied by a remodelling of cell membrane lipid composition [41]. This may be closely linked to the succession of microbial communities and adaptive changes to the environment [42]. In summary, KEGG enrichment analysis reveals signals for aromatic amino acid, alkaloid biosynthesis, and lipid metabolic remodelling, suggesting ecological adaptation between cell membrane lipids and aromatic metabolic networks [9,16,43].

From an animal nutrition and feed-safety perspective, several growth stage-associated metabolites warrant attention. Biogenic amines such as phenethylamine and agmatine were more pronounced at FS, which is consistent with greater protein decarboxylation and is generally considered a quality risk because biogenic amines may reduce palatability and can affect animal health at high intake. In contrast, betaine enrichment at MS may reflect osmoregulatory adaptation and could contribute to nutritional value as a methyl donor. Changes in free amino acids and their derivatives may influence the balance between rumen-degradable and rumen-undegradable nitrogen. Because this study used untargeted metabolomics with relative quantification and putative annotations, these nutritional interpretations should be confirmed by targeted quantification in future work.

### 4.4. Correlation Between Microorganisms, Metabolites and Phenotypes

By integrating correlations between microbial communities and metabolites (Figure 9), the roles of distinct functional microbial groups in shaping final silage quality were revealed. Beneficial lactic acid bacteria represented by Lactobacillus and Streptococcus exhibited positive correlations with multiple amino acid metabolites and negative correlations with BA. This indicates they are not only primary lactic acid producers but may also influence amino acid conversion pathways and inhibit BA growth through their metabolic activities [44]. Conversely, Enterococcus’s positive correlations with harmful metabolites (e.g., biogenic amines) and adverse fermentation indicators (BA, NH_3_-N/TN) designate it as an indicator organism for silage quality risks during the FS. Conditionally pathogenic bacteria like Enterobacter and Klebsiella showed positive correlations with certain lipids (e.g., LPC 18:2), potentially reflecting their activities in utilising membrane lipids or participating in lipid remodelling. These patterns align with concerns regarding ‘biogenic amines—palatability and health risks’ in silage food safety reviews [34,40], though their ecological functions warrant further investigation. Notably, the osmoprotectant betaine exhibited negative correlations with most bacteria. This may indicate that during late fermentation, as environmental stresses (such as low pH and low water activity) intensify, microbial demand for compatible solutes increases, yet their synthesis or accumulation is inhibited [45]. This negative relationship between metabolites and microorganisms reveals metabolic trade-offs undertaken by microbes to counter environmental stress during silage fermentation.

Since the experiment was conducted at a single location and year, using only one oat variety, the effects of climate change and variety were not evaluated. Secondly, laboratory vacuum-bagged silage may not fully represent large-scale silo silage, as there are differences in packing density gradients and the risk of oxygen ingress. Thirdly, untargeted metabolomics provides relative rather than absolute quantification, and metabolite annotation based on spectral libraries may include isomers or putative identifications. Therefore, key markers need to be validated in a targeted manner in future work.

## 5. Conclusions

This study demonstrates that the growth stage significantly influences fermentation quality, bacterial community composition, and metabolomic characteristics of oat silage. Based on the integrated assessment of fermentation indices, nutrient retention, microbiome, and metabolome, MS was the optimal harvest period for oat silage. Although DS silage showed Lactobacillus enrichment and comparatively lower NH_3_-N/TN, its higher fibre and lower CP constrained its overall nutritional value; therefore, supplemental sugars or targeted inoculation may be required to improve fermentation efficiency. For FS silage, wilting before ensiling is recommended to reduce moisture and suppress clostridial fermentation. Overall, the differences in silage quality at different stages mainly stem from the characteristics of the raw materials and the microorganisms initially attached to them, as well as the subsequent microbial succession process, which mediates fermentation.

This study employs multi-omics technologies to elucidate the intricate mechanisms governing silage quality at the microscopic level, providing robust theoretical foundations and practical guidance for precision production of oat silage.

## Figures and Tables

**Table 1:** Nutritional quality of fresh oats raw material and silage.

Items	FS		MS		DS	
	Raw Material	Silage	Raw Material	Silage	Raw Material	Silage
DM g/kg FM	232.62 ± 6.87 c	257.74 ± 7.62 C	297.94 ± 7.80 b	326.07 ± 11.40 B	369.73 ± 4.21 a	385.27 ± 4.05 A
CP g/kg DM	96.26 ± 2.48 a	84.46 ± 3.71 A	90.07 ± 1.85 b	87.17 ± 1.73 A	71.44 ± 1.05 c	69.97 ± 1.43 B
WSC g/kg DM	27.13 ± 1.32 c	7.49 ± 0.78 C	42.92 ± 1.99 b	12.75 ± 0.14 B	50.55 ± 1.02 a	21.44 ± 0.48 A
NDF g/kg DM	492.13 ± 4.52 c	457.01 ± 23.20 B	580.17 ± 6.85 b	524.51 ± 11.37 A	607.86 ± 11.89 a	563.52 ± 21.74 A
ADF g/kg DM	252.69 ± 12.24 b	201.90 ± 12.86 B	281.20 ± 5.50 ab	247.91 ± 8.95 A	300.28 ± 11.28 a	266.43 ± 17.62 A
Ash g/kg DM	43.98 ± 2.05 a	73.12 ± 2.11 A	44.35 ± 1.54 a	69.43 ± 4.38 A	43.36 ± 1.25 a	57.04 ± 1.38 B

Note: FS: Flowering stage, MS: Milk ripening stage, DS: Wax ripening Stage; FM: Fresh matter, DM: Dry matter, CP: Crude protein, WSC: Water-soluble carbohydrates, NDF: Neutral detergent fibre, ADF: Acid detergent fibre. Values are presented as mean ± standard error (*n* = 4); different letters indicate significant differences among treatments in the same row (*p* < 0.05), and the same applies below. Differences among fresh oat raw material treatments are indicated by lowercase letters (a, b, c), while differences among silage feed treatments are indicated by uppercase letters (A, B, C).

**Table 2:** Fermentation quality and microbial counts of oat silage.

Items	FS	MS	DS
pH	4.82 ± 0.06 ab	4.63 ± 0.08 b	4.89 ± 0.06 a
LA g/kg DM	14.96 ± 1.99 a	8.02 ± 1.20 b	8.49 ± 0.96 b
AA g/kg DM	0.48 ± 0.14 a	0.21 ± 0.06 a	0.22 ± 0.02 a
PA g/kg DM	10.13 ± 0.84 b	2.79 ± 1.22 c	14.66 ± 1.25 a
BA g/kg DM	31.13 ± 2.86 a	10.02 ± 2.86 b	12.20 ± 1.59 b
NH_3_-N/TN %	12.57 ± 0.31 a	7.02 ± 0.24 b	6.73 ± 0.20 b
LAB (log_10_cfu/g FM)	6.49 ± 0.06 a	4.49 ± 0.51 b	5.21 ± 0.41 b
EC (log_10_cfu/g FM)	ND	1.80 ± 0.92 a	0.17 ± 0.17 a
Yeast&Moulds (log_10_cfu/g FM)	5.70 ± 0.26 a	3.85 ± 0.24 b	3.41 ± 0.17 b

Note: FS: Flowering stage, MS: Milk ripening stage, DS: Wax ripening stage, DM: Dry matter, LA: Lactic acid, AA: Acetic acid, PA: Propionic acid, BA: Butyric acid, NH_3_-N/TN: Ammoniacal Nitrogen/Total Nitrogen, LAB: Lactic acid bacteria, EC: Escherichia coli. Values are presented as mean ± standard error (*n* = 4); different letters among data in the same row indicate significant differences between treatments (*p* < 0.05). ND, not detected.

## Data Availability

The original contributions presented in this study are included in the article. Further inquiries can be directed to the corresponding author.

## Supplementary Materials

The following supporting information can be downloaded at https://www.mdpi.com/article/10.3390/microorganisms14030516/s1. Supplementary materials on the clustering and number statistics of differential metabolites at three growth stages, as well as differential KEGG enrichment, can be found in the attachment. Table S1: Summary of differential analysis detected by LC-MS; Tables S2 and S3: Number statistics and significance lists of differential metabolites used for KEGG enrichment.
